# Comparative analysis of obesity-related cardiometabolic and renal biomarkers in human plasma and serum

**DOI:** 10.1038/s41598-019-51673-0

**Published:** 2019-10-28

**Authors:** Meenu Rohini Rajan, Matus Sotak, Fredrik Barrenäs, Tong Shen, Kamil Borkowski, Nicholas J. Ashton, Christina Biörserud, Tomas L. Lindahl, Sofia Ramström, Michael Schöll, Per Lindahl, Oliver Fiehn, John W. Newman, Rosie Perkins, Ville Wallenius, Stephan Lange, Emma Börgeson

**Affiliations:** 1 Department of Molecular and Clinical Medicine, Wallenberg Laboratory, Institute of Medicine, University of Gothenburg, Gothenburg, Sweden; 2 Wallenberg Centre for Molecular and Translational Medicine, University of Gothenburg, Gothenburg, Sweden; 3 Department of Cell & Molecular Biology, Uppsala University, Uppsala, Sweden; 4 NIH West Coast Metabolomics Center, Genome Center, University of California Davis, Davis, USA; 5 Department of Psychiatry and Neurochemistry, Institute of Physiology and Neuroscience, University of Gothenburg, Gothenburg, Sweden; 6 King’s College London, Institute of Psychiatry, Psychology & Neuroscience, Maurice Wohl Clinical Neuroscience Institute, London, UK; 7 NIHR Biomedical Research Centre for Mental Health & Biomedical Research Unit for Dementia at South London & Maudsley NHS Foundation, London, UK; 8 Dementia Research Centre, Institute of Neurology, University College London, London, UK; 9 Department of Gastrosurgical Research and Education, Institute of Clinical Sciences, Sahlgrenska University Hospital, University of Gothenburg, Gothenburg, Sweden; 10 Department of Clinical Chemistry and Department of Clinical and Experimental Medicine, Linköping University, Linköping, Sweden; 11 Cardiovascular Research Centre, School of Medical Sciences, Örebro University, Örebro, Sweden; 12 Department of Nutrition, University of California Davis, Davis, USA; 13 USDA, ARS, Western Human Nutrition Research Center, Davis, USA; 14 Division of Cardiology, School of Medicine, University of California San Diego, San Diego, USA; 15 Department of Clinical Physiology, Sahlgrenska University Hospital, Gothenburg, Sweden

**Keywords:** Translational immunology, Biomarkers, Metabolic disorders

## Abstract

The search for biomarkers associated with obesity-related diseases is ongoing, but it is not clear whether plasma and serum can be used interchangeably in this process. Here we used high-throughput screening to analyze 358 proteins and 76 lipids, selected because of their relevance to obesity-associated diseases, in plasma and serum from age- and sex-matched lean and obese humans. Most of the proteins/lipids had similar concentrations in plasma and serum, but a subset showed significant differences. Notably, a key marker of cardiovascular disease PAI-1 showed a difference in concentration between the obese and lean groups only in plasma. Furthermore, some biomarkers showed poor correlations between plasma and serum, including PCSK9, an important regulator of cholesterol homeostasis. Collectively, our results show that the choice of biofluid may impact study outcome when screening for obesity-related biomarkers and we identify several markers where this will be the case.

## Introduction

Obesity-related illness is an increasingly important global health issue that places a tremendous economic burden on society^1^. The negative health effects of prolonged obesity are partly fuelled by chronic low-grade inflammation, which contributes to cardiometabolic and kidney pathophysiology^2–4^. However, the exact mechanisms that link obesity with cardiometabolic and kidney diseases are unclear and remain a subject of intensive research. The search for biomarkers that assist in the identification of novel disease-related pathways is critical to develop new therapies that are tailored to subpopulations particularly prone to obesity-related pathophysiology.

Disease-related biomarkers are often identified and quantified in blood-derived plasma or serum^5,6^. Preparation of plasma and serum requires the removal of cellular components by centrifugation. Generation of plasma is preceded by the addition of an anti-coagulant (e.g. EDTA, heparin or citrate) to the whole blood. By contrast, the blood used for serum is allowed to clot before centrifugation, resulting in lower concentrations of clotting factors (such as fibrinogen and coagulation cascade proteins) in serum than in plasma. The World Health Organization generally recommends using plasma as this more accurately reflects the physiological and/or pathophysiological state of the patient^7^. However, biomarkers are often reported to have better detectability in serum^8^ despite the fact that serum has a slightly lower total protein concentration than plasma^9^. Indeed, some intracellularly stored proteins and lipids are only detectable upon coagulation-induced release from leukocytes and platelets, and serum is preferred in assays detecting, for example, cardiac troponins^10–12^. Importantly, the choice of biofluid is not merely a question of detectability, but it may also affect the conclusions drawn from a study. For example, Alsaif *et al*. showed that of 16 proteins (identified in either plasma or serum) that were differentially expressed between healthy controls and subjects with bipolar disorder, only two showed differential expression in both serum and plasma^13^.

The aim of our study was to determine whether the use of plasma or serum would yield different results when screening for obesity-related biomarkers. We analyzed proteins and lipids that have previously been suggested to play a role in obesity-related cardiometabolic diseases in plasma and serum from age- and sex-matched groups of lean and obese humans. Our results show that the use of plasma or serum may have an effect on study outcome when screening for obesity-related biomarkers and we identify key markers that highlight this issue.

## Results and Discussion

### Detectability of proteins in plasma versus serum

We used four Olink multiplex protein panels (inflammation, cardiometabolic, cardiovascular II, cardiovascular III) selected on the basis of their relevance to obesity-related diseases to measure protein concentrations in plasma and serum from 11 obese subjects and 11 age- and sex-matched lean controls. The characteristics of the human cohort are presented in Table 1. Of the 368 proteins analyzed (10 of which were measured in duplicate panels, see Supplementary Table S1 for the full list), one protein (BDNF) was excluded due to technical issues, nine proteins (IL-1 alpha, IL-2, TSLP, IL-22 RA1, IL-13, TNF, IL-20, IL-33, IFN-gamma) were excluded because they were undetectable in both plasma and serum, and 23 additional proteins were excluded because values were missing in >30% of the samples in all of the four groups (lean plasma, lean serum, obese plasma, obese serum; Supplementary Table S1). Detectability issues with one of the excluded proteins, NT-proBNP, have previously been reported^14^. In total, 335 proteins were included in the comparative analyses (Supplementary Fig. S1**)**.

**Table 1:** Summary of cohort demographics.

	Lean			Obese		
	Men	Women	All	Men	Women	All
Sex	3	8	3♂/8♀	3	8	3♂/8♀
BMI (kg/m^2^)	23.3 ± 0.9	22.0 ± 1.0	22.4 ± 2.4	41.0 ± 2.1	44.4 ± 1.5	43.5 ± 4.1
Age (years)	42.3 ± 4.3	40.0 ± 5.3	40.6 ± 13.0	45.0 ± 3.1	40.4 ± 5.6	41.6 ± 13.6
Hormone replacement therapy	—	0/8	—	—	0/8	—
Hormonal contraceptive pill	—	2/8	—	—	0/8	—
Intrauterine contraceptive device	—	0/8	—	—	2/8	—

Data are shown as mean ± SEM.

**Figure 1:**
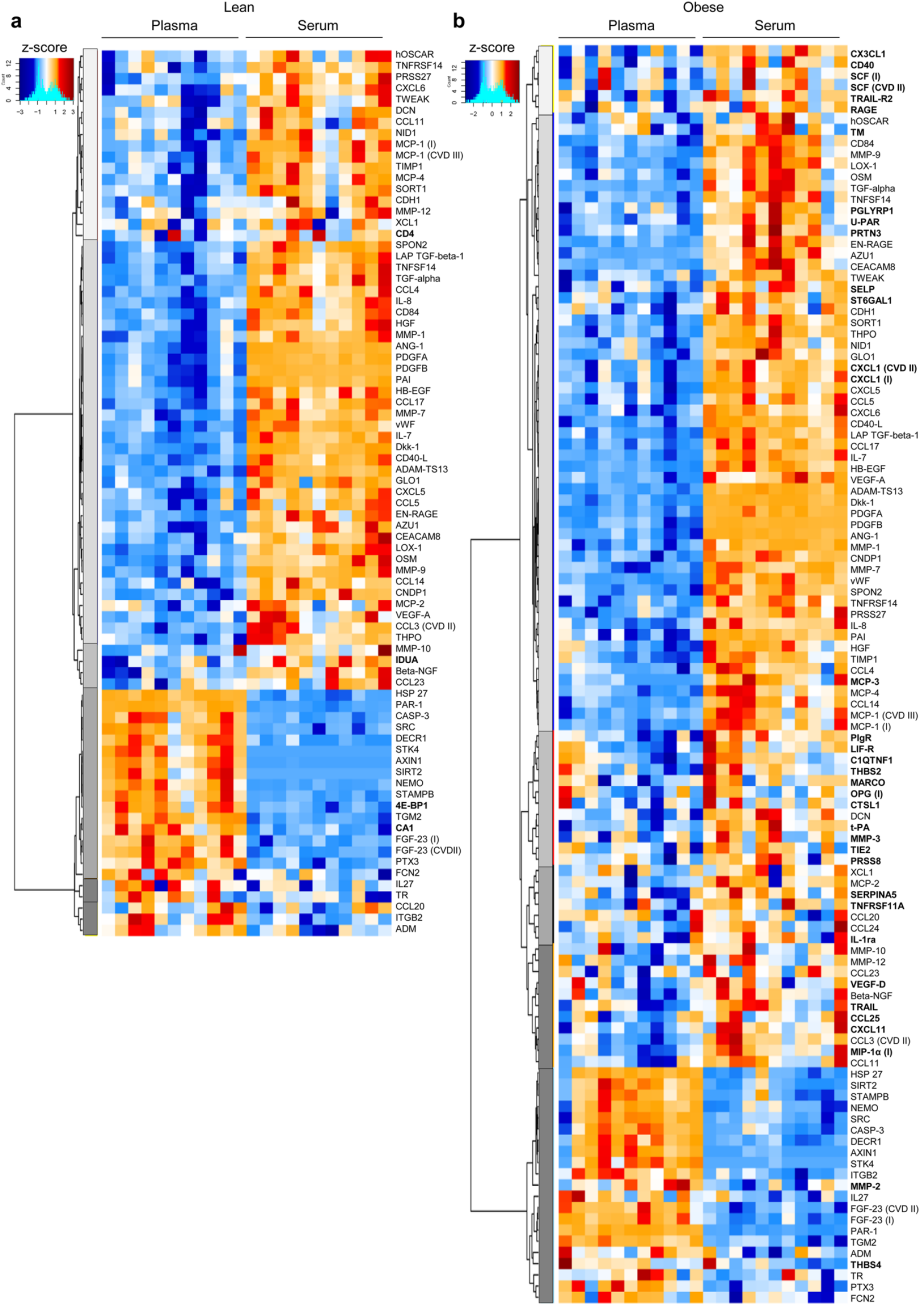
Detectability of proteins in plasma versus serum. Heatmaps showing protein biomarkers that exhibited significantly different concentrations in plasma versus serum in (**a**) lean subjects (n = 11) and (**b**) obese subjects (n = 11) after adjustment for multiple comparisons using the method of Holm-Bonferroni at adj. p < 0.05. Proteins that are significantly different in only one of the groups (lean or obese) are marked in bold. For proteins that are present in duplicate protein panels, the panel is indicated in parentheses: I, inflammation; CVII, cardiovascular II; and CVIII, cardiovascular III. Relative protein concentrations are reported as z-scores.

For the majority of proteins, their concentrations were similar between plasma and serum (Supplementary Fig. S2a,b). After adjusting for multiple comparisons using the stringent Holm-Bonferroni test, we found significantly different concentrations between plasma and serum for 23.5% and 33.4% of proteins in the lean and obese cohorts, respectively [adjusted (adj.) p < 0.05, Fig. 1]. Most of these proteins were present at higher concentrations in serum, which may partly be explained by the clotting-induced volume displacement effect^15,16^ and by the fact that coagulation elicits release of platelet granules and intracellularly stored cytokines^17–19^. The intracellularly stored protein MCP-1, for example, exhibited significantly higher concentrations in serum compared with plasma (in both the inflammation and the cardiovascular III panels) in the lean and obese groups. Of note, we did not record female menstruation cycle and/or menopausal state, which may affect platelet activation, although conflicting results have been shown^20–25^.

A subset of proteins with significantly different concentrations in plasma and serum (including HSP-27, PAR-1, 4E-BP1 and SRC) exhibited lower concentrations in serum (Fig. 1). HSP-27 has been proposed as a biomarker for both cardiometabolic disease and cancer^26^, although controversial results have been reported^27^. Of note, a recent study showed that the concentration of HSP-27 increased by about three-fold with just one freeze-thaw cycle in plasma but was more stable in serum^28^. All of our samples underwent two freeze-thaw cycles, which could explain the higher detectability of HSP-27 in plasma. During coagulation, PAR-1 is cleaved^29^ and SRC^30^ and 4-EBP1^31,32^ become prone to degradation through proteolytic pathways, which likely explains their lower concentrations in serum. Furthermore, two proteins, AXIN1 and STK4, passed our cut-off criteria for detection in plasma but not in serum (Supplementary Table S1). Enrichment analysis of all the proteins that were significantly altered between plasma and serum confirmed the enrichment of pathways involved in neutrophil chemotaxis and platelet activation (Supplementary Fig. S2c, Supplementary Table S3).

### Sensitivity of plasma versus serum when screening for obesity-related protein biomarkers

Most of the biomarkers that showed significantly different concentrations between the obese and lean groups were present at higher levels in the obese group; however, a small number (including IGFBP-1 and GH) showed lower concentrations in the obese group, with significant differences observed in both plasma and serum (Table 2). The number of proteins with significantly different concentrations between the lean and obese groups was greater in serum (Table 2), in agreement with (1) an earlier study that reported higher sensitivity of serum to detect diabetes-associated differences in metabolite concentration^33^ and (2) the fact that obesity is associated with higher leukocyte and platelet counts and increased platelet activation^34,35^. MCP-3 was present at higher concentrations in the obese versus lean group in serum but not plasma (Table 2), and showed low detectability in plasma in both groups (Supplementary Table S1). However, concentrations of PAI-1 were only significantly higher in the obese versus lean group in plasma despite showing higher detectability in serum (Table 2). This difference in sensitivity versus detectability for PAI-1 was confirmed by ELISA (Supplementary Fig. S3). PAI-1 inhibits fibrinolysis and has been proposed to be an important biomarker in cardiometabolic and diabetes research, although, as recently reviewed, conflicting results have been reported^36^. A possible explanation for this discrepancy, at least in part, may be due to the interchangeable use of plasma versus serum; indeed, studies comparing lean versus obese and/or diabetic groups have reported differences in PAI-1 levels when using plasma^37^ but not serum^38^. Coagulation-induced secretion of intracellular PAI-1 is likely responsible for the high serum levels of PAI-1, which may mask the differences between the lean and obese groups.

**Table 2:** Proteins that exhibited significant differences in concentrations between obese and lean groups in plasma and/or serum.

Protein	Plasma					Serum				
	Lean	Obese	Obese vs lean log_2_ ratio	H-B Adj. p value	FDR Adj. p value	Lean	Obese	Obese vs lean log_2_ ratio	H-B Adj. p value	FDR Adj. p value
	NPX (mean ± SEM)					NPX (mean ± SEM)				
4E-BP1	6.57 ± 0.21	7.54 ± 0.10	0.97	ns	0.0106	4.57 ± 0.10	6.18 ± 0.33	1.62	ns	0.0081
ADAM-TS13	5.17 ± 0.06	4.94 ± 0.04	−0.22	ns	ns	6.38 ± 0.05	6.19 ± 0.03	−0.18	ns	0.0336
ADM	6.33 ± 0.06	7.11 ± 0.09	0.78	0.0002	0.0001	6.03 ± 0.08	6.82 ± 0.11	0.79	0.0032	0.0004
AGRP	3.64 ± 0.11	3.12 ± 0.07	−0.52	ns	0.0136	3.74 ± 0.13	3.10 ± 0.08	−0.64	ns	0.0081
AMBP	5.52 ± 0.02	5.72 ± 0.04	0.20	ns	0.0037	5.53 ± 0.05	5.78 ± 0.03	0.26	ns	0.0058
CCL3 (CVD II)	3.18 ± 0.04	3.67 ± 0.05	0.49	0.0002	0.0001	3.65 ± 0.07	4.13 ± 0.07	0.48	0.0241	0.0018
CCL4	6.04 ± 0.07	6.67 ± 0.13	0.63	ns	0.0095	7.33 ± 0.12	7.76 ± 0.19	0.43	ns	ns
CCL18	5.28 ± 0.22	6.09 ± 0.19	0.81	ns	ns	5.36 ± 0.21	6.25 ± 0.18	0.89	ns	0.0259
CCL19	8.98 ± 0.15	9.66 ± 0.13	0.68	ns	0.0246	9.16 ± 0.15	9.82 ± 0.12	0.66	ns	0.0222
CDCP1	1.56 ± 0.14	2.16 ± 0.17	0.60	ns	ns	1.64 ± 0.14	2.37 ± 0.18	0.73	ns	0.0274
CES1	1.41 ± 0.05	2.04 ± 0.20	0.63	ns	ns	1.31 ± 0.04	2.02 ± 0.16	0.71	ns	0.0136
CHI3L1	5.27 ± 0.21	6.21 ± 0.27	0.94	ns	ns	5.60 ± 0.13	6.61 ± 0.27	1.01	ns	0.0266
CHL1	2.47 ± 0.09	2.14 ± 0.04	−0.33	ns	0.0406	2.64 ± 0.12	2.20 ± 0.05	−0.43	ns	0.0366
CSF-1	7.03 ± 0.06	7.30 ± 0.07	0.27	ns	ns	7.13 ± 0.05	7.43 ± 0.08	0.30	ns	0.0349
CSTB	3.65 ± 0.14	4.27 ± 0.12	0.62	ns	0.0321	3.65 ± 0.13	4.54 ± 0.21	0.89	ns	0.0155
CTSD	3.76 ± 0.09	4.61 ± 0.11	0.85	0.0025	0.0003	4.14 ± 0.05	4.94 ± 0.14	0.79	0.0400	0.0026
CTSZ	3.81 ± 0.13	4.33 ± 0.12	0.52	ns	ns	3.98 ± 0.06	4.46 ± 0.14	0.47	ns	0.0373
CXCL10	8.82 ± 0.17	9.51 ± 0.15	0.69	ns	ns	8.70 ± 0.18	9.59 ± 0.15	0.89	ns	0.0127
CXCL11	6.36 ± 0.16	6.96 ± 0.18	0.60	ns	ns	6.87 ± 0.16	7.94 ± 0.26	1.07	ns	0.0213
ENG	1.47 ± 0.07	1.40 ± 0.06	−0.07	ns	ns	1.54 ± 0.05	1.31 ± 0.03	−0.23	ns	0.0183
FABP4	3.65 ± 0.30	5.57 ± 0.15	1.93	0.0115	0.0010	3.78 ± 0.26	5.72 ± 0.16	1.93	0.0024	0.0004
FCN2	4.48 ± 0.16	5.22 ± 0.10	0.74	ns	0.0106	4.04 ± 0.14	4.87 ± 0.10	0.83	0.0422	0.0026
FGF-21 (CVD II)	4.56 ± 0.43	7.12 ± 0.48	2.56	ns	0.0105	4.54 ± 0.43	7.03 ± 0.48	2.49	ns	0.0105
FGF-21 (I)	3.50 ± 0.41	5.98 ± 0.44	2.48	ns	0.0086	3.59 ± 0.40	6.03 ± 0.44	2.44	ns	0.0081
Gal-9	6.96 ± 0.04	7.51 ± 0.07	0.55	0.0019	0.0003	7.07 ± 0.06	7.64 ± 0.08	0.57	0.0045	0.0005
GH	9.51 ± 0.69	6.38 ± 0.66	−3.13	ns	0.0318	9.63 ± 0.69	6.46 ± 0.64	−3.17	ns	0.0222
GLO1	3.29 ± 0.10	3.78 ± 0.18	0.49	ns	ns	4.66 ± 0.17	5.56 ± 0.25	0.90	ns	0.0415
HAOX1	2.90 ± 0.30	4.71 ± 0.41	1.80	ns	0.0220	2.97 ± 0.31	4.81 ± 0.42	1.84	ns	0.0183
HB-EGF	3.84 ± 0.09	3.94 ± 0.08	0.10	ns	ns	5.32 ± 0.13	6.52 ± 0.16	1.20	0.0030	0.0004
HGF	6.76 ± 0.07	7.62 ± 0.14	0.86	0.0249	0.0019	7.58 ± 0.09	8.60 ± 0.14	1.02	0.0026	0.0004
IGFBP-1	3.85 ± 0.20	1.47 ± 0.26	−2.38	0.0002	0.0001	3.98 ± 0.18	1.55 ± 0.27	−2.44	0.0002	0.0002
IGFBP-2	6.65 ± 0.25	5.81 ± 0.12	−0.85	ns	ns	6.84 ± 0.21	5.95 ± 0.11	−0.89	ns	0.0146
IL-1ra	5.33 ± 0.09	7.05 ± 0.21	1.72	0.0013	0.0003	5.80 ± 0.10	7.47 ± 0.20	1.67	0.0009	0.0004
IL-6	2.36 ± 0.17	4.17 ± 0.34	1.81	ns	0.0044	2.46 ± 0.16	4.22 ± 0.33	1.75	ns	0.0043
IL-10RB	6.34 ± 0.09	6.70 ± 0.08	0.36	ns	0.0473	6.53 ± 0.08	6.94 ± 0.08	0.41	ns	0.0146
IL-18	7.75 ± 0.14	8.47 ± 0.20	0.72	ns	ns	7.88 ± 0.15	8.67 ± 0.22	0.79	ns	0.0396
IL-18R1	6.61 ± 0.11	7.16 ± 0.13	0.55	ns	0.0360	6.80 ± 0.09	7.37 ± 0.13	0.57	ns	0.0188
KIT	3.31 ± 0.08	2.84 ± 0.09	−0.47	ns	0.0095	3.29 ± 0.08	2.99 ± 0.10	−0.30	ns	ns
LAP TGF-β−1	5.64 ± 0.11	6.01 ± 0.08	0.38	ns	ns	6.94 ± 0.09	7.28 ± 0.08	0.34	ns	0.0417
LEP	4.06 ± 0.32	6.66 ± 0.11	2.60	0.0019	0.0003	4.09 ± 0.34	6.81 ± 0.11	2.72	0.0018	0.0004
LILRB2	2.18 ± 0.09	2.62 ± 0.08	0.44	ns	0.0225	2.11 ± 0.11	2.68 ± 0.08	0.58	ns	0.0083
LTBR	1.75 ± 0.12	2.02 ± 0.08	0.27	ns	ns	1.84 ± 0.03	2.10 ± 0.05	0.26	ns	0.0043
MCP-1	9.35 ± 0.07	9.87 ± 0.05	0.52	0.0058	0.0005	10.52 ± 0.13	11.02 ± 0.13	0.51	ns	ns
MCP-3	1.39 ± 0.00	1.48 ± 0.04	0.09	ns	ns	1.42 ± 0.02	2.07 ± 0.10	0.65	0.0254	0.0018
MCP-4	2.19 ± 0.16	2.72 ± 0.12	0.53	ns	ns	3.56 ± 0.17	4.36 ± 0.22	0.80	ns	0.0450
MIP-1 alpha (I)	3.35 ± 0.03	3.83 ± 0.06	0.48	0.0027	0.0003	3.72 ± 0.07	4.28 ± 0.08	0.56	0.0107	0.0009
MPO	2.30 ± 0.23	2.91 ± 0.08	0.61	ns	ns	2.92 ± 0.13	3.52 ± 0.12	0.59	ns	0.0222
NCAM1	2.27 ± 0.09	1.89 ± 0.06	−0.38	ns	0.0191	2.26 ± 0.10	1.90 ± 0.08	−0.36	ns	ns
NEMO	3.40 ± 0.17	3.76 ± 0.22	0.36	ns	ns	1.58 ± 0.04	2.27 ± 0.18	0.69	ns	0.0223
OSM	2.40 ± 0.12	3.39 ± 0.21	1.00	ns	0.0106	3.95 ± 0.16	5.14 ± 0.29	1.19	ns	0.0208
PAI-1	3.49 ± 0.31	5.80 ± 0.23	2.31	0.0030	0.0003	7.21 ± 0.09	7.60 ± 0.07	0.39	ns	0.0274
PLC	5.00 ± 0.14	5.45 ± 0.07	0.45	ns	ns	5.23 ± 0.06	5.61 ± 0.03	0.38	0.0064	0.0006
PON3	5.45 ± 0.26	4.29 ± 0.27	−1.17	ns	0.0406	5.43 ± 0.21	4.22 ± 0.26	−1.22	ns	0.0146
PRCP	0.78 ± 0.07	1.11 ± 0.06	0.33	ns	0.0231	0.68 ± 0.05	1.11 ± 0.06	0.43	0.0067	0.0006
PRSS8	8.75 ± 0.08	9.20 ± 0.08	0.45	ns	0.0106	8.93 ± 0.09	9.43 ± 0.08	0.50	ns	0.0073
RARRES2	9.60 ± 0.13	10.22 ± 0.07	0.62	ns	0.0106	9.97 ± 0.09	10.44 ± 0.05	0.48	ns	0.0036
SCGB3A2	2.12 ± 0.26	0.96 ± 0.12	−1.15	ns	0.0130	2.23 ± 0.26	0.98 ± 0.13	−1.25	ns	0.0081
SELE	2.10 ± 0.13	2.81 ± 0.12	0.70	ns	0.0095	2.26 ± 0.12	2.93 ± 0.14	0.67	ns	0.0146
SPON2	9.78 ± 0.04	10.01 ± 0.04	0.24	ns	0.0036	10.31 ± 0.05	10.54 ± 0.03	0.23	ns	0.0105
STAMPB	3.16 ± 0.12	3.56 ± 0.15	0.41	ns	ns	1.84 ± 0.04	2.38 ± 0.16	0.54	ns	0.0450
t-PA	4.06 ± 0.20	5.23 ± 0.09	1.17	0.0276	0.0019	4.28 ± 0.24	5.89 ± 0.09	1.61	0.0082	0.0007
TGM2	6.03 ± 0.13	6.35 ± 0.08	0.32	ns	ns	3.85 ± 0.08	4.66 ± 0.17	0.81	ns	0.0082
TNF-R1	4.69 ± 0.14	5.27 ± 0.06	0.58	ns	0.0220	5.01 ± 0.06	5.53 ± 0.06	0.52	0.0019	0.0004
TNF-R2	3.24 ± 0.13	3.64 ± 0.04	0.41	ns	ns	3.42 ± 0.07	3.77 ± 0.07	0.35	ns	0.0146
TNFRSF10A	2.06 ± 0.05	2.27 ± 0.07	0.22	ns	ns	2.13 ± 0.07	2.42 ± 0.07	0.29	ns	0.0462
TNFRSF11A	4.04 ± 0.09	4.60 ± 0.08	0.56	ns	0.0036	4.40 ± 0.12	4.94 ± 0.08	0.54	ns	0.0146
TNFSF14	2.96 ± 0.08	3.73 ± 0.08	0.77	0.0004	0.0001	4.23 ± 0.12	4.97 ± 0.19	0.74	ns	0.0274
TR-AP	3.40 ± 0.14	3.89 ± 0.08	0.49	ns	ns	3.54 ± 0.14	4.12 ± 0.10	0.58	ns	0.0266
TRAIL-R2	4.40 ± 0.09	4.66 ± 0.05	0.26	ns	ns	4.59 ± 0.09	4.89 ± 0.05	0.30	ns	0.0481
TRAIL	7.25 ± 0.09	7.61 ± 0.08	0.36	ns	ns	7.49 ± 0.09	7.92 ± 0.09	0.43	ns	0.0213
U-PAR	3.28 ± 0.15	3.64 ± 0.06	0.36	ns	ns	3.78 ± 0.07	4.26 ± 0.10	0.48	ns	0.0104
VEGF-A	9.19 ± 0.06	9.58 ± 0.05	0.40	0.0196	0.0016	9.85 ± 0.13	10.40 ± 0.09	0.55	ns	0.0213
vWF	3.18 ± 0.18	3.55 ± 0.11	0.37	ns	ns	6.21 ± 0.15	6.95 ± 0.18	0.73	ns	0.0349

Differences in mean normalized protein expression (NPX) values between obese and lean groups are reported as a log_2_ ratio. p values were adjusted for multiple comparisons using either Holm-Bonferroni (H-B) or false discovery rate (FDR); ns, not significant (adj. p > 0.05). For proteins that are present in duplicate protein panels, the panel is indicated in parentheses: I, inflammation; CVII, cardiovascular II; and CVIII, cardiovascular III.

### Protein correlations in plasma versus serum

For the correlation analysis, 316 proteins survived the cut-off criteria (Supplementary Fig. S1**)**. We observed significant correlations between plasma and serum samples for most (68.8%) of the proteins analyzed in the lean and obese groups combined (Table 3), although fewer significant correlations were seen when dividing the cohort into obese and lean (Supplementary Table S4). Of the 10 proteins that were measured in duplicate panels, eight displayed similar correlations between plasma and serum. However, MCP-1 and uPA only showed a significant correlation between plasma and serum in one of the duplicate panels.

**Table 3:** Correlations of protein concentrations in plasma versus serum in all subjects.

Inflammation			Cardiometabolic			Cardiovascular II			Cardiovascular III		
Protein	r	H-B Adj. p value	Protein	r	H-B Adj. p value	Protein	r	H-B Adj. p value	Protein	r	H-B Adj. p value
FGF-21	1.00	1.57E-20	MBL2	0.99	2.79E-15	GH	1.00	1.47E-25	IGFBP-1	0.98	4.93E-13
FGF-19	0.99	1.11E-15	FCGR2A	0.98	6.92E-14	FGF-21	1.00	1.82E-22	Ep-CAM	0.97	1.47E-11
CCL20	0.99	7.03E-15	LILRB5	0.97	5.69E-12	LEP	1.00	2.85E-21	FABP4	0.96	1.32E-10
IL-18	0.98	3.69E-14	FCN2	0.97	9.41E-11	HAOX1	1.00	8.14E-20	CHIT1	0.96	1.85E-10
MMP-10	0.98	1.01E-13	LYVE1	0.96	6.02E-10	SERPINA12	0.99	1.00E-17	TFF3	0.96	2.79E-10
CXCL9	0.98	2.95E-13	CCL18	0.95	1.43E-09	IL-6	0.99	1.90E-16	SCGB3A2	0.95	3.62E-09
CDCP1	0.97	2.68E-11	COMP	0.95	6.47E-09	FABP2	0.99	8.91E-16	IGFBP-2	0.94	2.47E-08
TRANCE	0.97	6.59E-11	TIMD4	0.95	9.36E-09	KIM-1	0.99	2.38E-14	CCL24	0.94	2.85E-08
MCP-2	0.97	1.05E-10	THBS4	0.94	1.10E-08	IL-18	0.98	3.44E-13	PON3	0.94	3.53E-08
CCL19	0.96	4.09E-10	IGLC2	0.94	2.73E-08	GIF	0.98	1.35E-12	TR	0.93	9.55E-08
IL-12B	0.96	1.30E-09	REG1A	0.94	3.16E-08	CTRC	0.98	2.26E-12	CCL22	0.92	1.88E-07
OPG	0.95	4.06E-09	CR2	0.94	3.88E-08	MMP-12	0.98	4.02E-12	t-PA	0.92	2.80E-07
PD-L1	0.95	4.80E-09	FCGR3B	0.93	5.93E-08	REN	0.97	1.18E-11	CPA1	0.92	5.11E-07
CXCL10	0.95	9.69E-09	PRSS2	0.93	7.84E-08	IL-1ra	0.97	1.28E-11	DLK-1	0.91	8.47E-07
IL-18R1	0.94	1.68E-08	ANGPTL3	0.93	1.56E-07	SCF	0.97	4.73E-11	CPB1	0.91	9.00E-07
Flt3L	0.93	7.43E-08	SAA4	0.93	1.61E-07	ADM	0.96	1.70E-10	TNFRSF10C	0.90	1.99E-06
uPA	0.93	8.58E-08	TNC	0.92	2.47E-07	ACE2	0.96	1.05E-09	CHI3L1	0.89	4.47E-06
CD6	0.93	1.38E-07	NRP1	0.92	2.75E-07	LPL	0.95	2.47E-09	CCL15	0.89	6.88E-06
SCF	0.93	1.45E-07	DPP4	0.92	5.55E-07	MMP-7	0.95	2.92E-09	MMP-3	0.89	7.27E-06
CCL23	0.92	2.06E-07	CRTAC1	0.90	2.52E-06	PRSS8	0.95	3.92E-09	TIMP4	0.88	1.43E-05
TNFB	0.92	2.87E-07	APOM	0.89	5.45E-06	XCL1	0.95	7.68E-09	LDL receptor	0.87	2.59E-05
TRAIL	0.91	6.72E-07	GP1BA	0.89	6.21E-06	VEGF-D	0.95	8.75E-09	SELE	0.87	3.29E-05
CD244	0.91	6.72E-07	LILRB2	0.89	7.09E-06	TNFRSF13B	0.94	1.13E-08	ST2	0.84	0.0002
CCL25	0.90	2.94E-06	FETUB	0.89	8.64E-06	HO-1	0.94	1.23E-08	IL-6RA	0.84	0.0002
OSM	0.87	2.65E-05	CDH1	0.88	1.36E-05	BMP-6	0.93	6.58E-08	CTSZ	0.83	0.0003
CCL11	0.87	3.92E-05	TIE1	0.88	1.95E-05	IgG Fc R II-b	0.93	7.84E-08	SHPS-1	0.81	0.0008
CST5	0.86	5.29E-05	NCAM1	0.87	2.06E-05	IL16	0.93	8.80E-08	CTSD	0.81	0.0009
CCL28	0.85	0.0001	TCN2	0.87	2.34E-05	RAGE	0.93	1.07E-07	CCL16	0.80	0.001
IL-10RB	0.85	0.0001	AOC3	0.87	3.12E-05	TIE2	0.93	1.26E-07	GDF-15	0.80	0.001
HGF	0.85	0.0001	VCAM1	0.85	0.0001	MERTK	0.92	3.06E-07	Gal-4	0.79	0.002
CCL4	0.84	0.0001	TGFBI	0.84	0.0002	TF	0.92	4.20E-07	CD93	0.79	0.002
TNFRSF9	0.84	0.0002	F7	0.84	0.0002	TRAIL-R2	0.92	4.51E-07	CD163	0.78	0.003
CSF-1	0.83	0.0003	C2	0.84	0.0002	IL27	0.92	4.63E-07	RARRES2	0.77	0.004
IL-8	0.83	0.0004	ANG	0.84	0.0002	Gal-9	0.91	6.07E-07	IL2-RA	0.75	0.007
MIP-1α	0.82	0.0005	SERPINA7	0.83	0.0004	IL1RL2	0.91	9.77E-07	RETN	0.75	0.008
CXCL11	0.79	0.002	OSMR	0.83	0.0004	AGRP	0.91	1.06E-06	BLM hydrol.	0.75	0.008
ADA	0.78	0.003	IGFBP6	0.82	0.0005	CTSL1	0.91	1.53E-06	MPO	0.74	0.009
TWEAK	0.78	0.003	ICAM3	0.81	0.0008	TNFRSF11A	0.90	2.86E-06	IL-17RA	0.73	0.01
MCP-4	0.78	0.003	PROC	0.81	0.0008	CD4	0.89	7.67E-06	TNF-R1	0.73	0.01
CD5	0.77	0.004	ICAM1	0.80	0.001	TM	0.88	1.09E-05	ICAM-2	0.73	0.01
CD40	0.77	0.004	QPCT	0.79	0.002	MARCO	0.88	1.12E-05	TLT-2	0.73	0.02
4E-BP1	0.76	0.006	PRCP	0.79	0.002	FS	0.88	1.15E-05	IL-18BP	0.72	0.02
LIF-R	0.76	0.007	IL7R	0.79	0.002	DCN	0.87	3.66E-05	IL-1RT2	0.72	0.02
DNER	0.75	0.007	C1QTNF1	0.78	0.003	SOD2	0.86	5.53E-05	PI3	0.71	0.02
IL-10	0.75	0.008	CHL1	0.78	0.003	CCL3	0.85	9.10E-05	PAI-1	0.71	0.02
MMP-1	0.73	0.01	SERPINA5	0.77	0.004	hOSCAR	0.84	0.0002	COL1A1	0.71	0.02
MCP-1	0.70	0.03	SPARCL1	0.77	0.004	PD-L2	0.83	0.0004	MEPE	0.71	0.02
TNFSF14	0.70	0.03	IGFBP3	0.76	0.005	THBS2	0.82	0.0005	TFPI	0.71	0.02
EN-RAGE	0.69	0.04	NID1	0.76	0.006	PlGF	0.82	0.0005	OPG	0.70	0.03
β-NGF	0.69	0.04	SELL	0.76	0.007	Protein BOC	0.82	0.0005	MB	0.69	0.04
VEGF-A	0.68	0.048	PCOLCE	0.75	0.008	PAR-1	0.81	0.0009	TR-AP	0.69	0.04
SLAMF1	0.65	ns	CST3	0.75	0.008	PRELP	0.77	0.004	PLC	0.64	ns
CXCL6	0.62	ns	CD59	0.74	0.01	AMBP	0.76	0.005	vWF	0.64	ns
LAP TGF-β-1	0.59	ns	GAS6	0.74	0.01	SORT1	0.76	0.006	MMP-9	0.63	ns
CXCL1	0.56	ns	CFHR5	0.73	0.01	VSIG2	0.76	0.006	CDH5	0.63	ns
CXCL5	0.56	ns	ST6GAL1	0.72	0.02	SPON2	0.73	0.01	PSP-D	0.63	ns
CX3CL1	0.55	ns	LILRB1	0.71	0.03	CCL17	0.73	0.01	uPA	0.62	ns
STAMPB	0.47	ns	F11	0.64	ns	CD84	0.70	0.03	GRN	0.62	ns
FGF-23	0.43	ns	CA4	0.62	ns	THPO	0.64	ns	ITGB2	0.62	ns
FGF-5	0.42	ns	TIMP1	0.62	ns	IDUA	0.64	ns	Gal-3	0.61	ns
CASP-8	0.25	ns	LCN2	0.62	ns	FGF-23	0.63	ns	AXL	0.60	ns
ST1A1	0.23	ns	PAM	0.56	ns	GLO1	0.63	ns	PGLYRP1	0.60	ns
IL-7	0.18	ns	VASN	0.54	ns	PSGL-1	0.61	ns	CSTB	0.59	ns
TGF-α	0.18	ns	KIT	0.52	ns	CXCL1	0.60	ns	PECAM-1	0.59	ns
ARTN	#	#	CNDP1	0.51	ns	PRSS27	0.53	ns	TNF-R2	0.58	ns
AXIN1	#	#	TNXB	0.51	ns	ANG-1	0.52	ns	U-PAR	0.58	ns
GDNF	#	#	ENG	0.50	ns	LOX-1	0.52	ns	TNFRSF14	0.58	ns
IL-10RA	#	#	MET	0.49	ns	ADAM-TS13	0.48	ns	CNTN1	0.56	ns
IL-15RA	#	#	GNLY	0.48	ns	TGM2	0.48	ns	Notch 3	0.53	ns
IL-17A	#	#	TGFBR3	0.48	ns	PTX3	0.48	ns	FAS	0.53	ns
IL-17C	#	#	CD46	0.47	ns	CEACAM8	0.44	ns	IGFBP-7	0.52	ns
IL-20RA	#	#	CA1	0.45	ns	GDF-2	0.42	ns	TNFSF13B	0.51	ns
IL-22RA1	#	#	PLXNB2	0.44	ns	CD40-L	0.39	ns	IL-1RT1	0.51	ns
IL-24	#	#	EFEMP1	0.43	ns	Dkk-1	0.36	ns	AP-N	0.51	ns
IL-2RB	#	#	CCL14	0.39	ns	HB-EGF	0.31	ns	SELP	0.50	ns
IL-4	#	#	CA3	0.38	ns	PDGF-B	0.31	ns	CXCL16	0.49	ns
IL-5	#	#	COL18A1	0.36	ns	SRC	0.26	ns	MMP-2	0.49	ns
IL-6	#	#	NOTCH1	0.35	ns	PIgR	0.24	ns	OPN	0.46	ns
LIF	#	#	PTPRS	0.32	ns	IL-17D	0.24	ns	PRTN3	0.44	ns
MCP-3	#	#	CCL5	0.22	ns	HSP 27	0.20	ns	LTBR	0.38	ns
NRTN	#	#	MFAP5	0.21	ns	BNP	#	#	SPON1	0.36	ns
NT3	#	#	MEGF9	0.11	ns	CA5A	#	#	MCP-1	0.34	ns
SIRT2	#	#	CES1	#	#	DECR1	#	#	ALCAM	0.28	ns
IFN-γ	nd	nd	DEFA1	#	#	GT	#	#	PCSK9	0.27	ns
IL-1α	nd	nd	FAP	#	#	IL-4RA	#	#	JAM-A	0.24	ns
IL-13	nd	nd	ITGAM	#	#	ITGB1BP2	#	#	CASP-3	0.20	ns
IL-2	nd	nd	LTBP2	#	#	NEMO	#	#	PDGF-A	0.16	ns
IL-20	nd	nd	PLA2G7	#	#	PAPPA	#	#	KLK6	0.15	ns
IL-33	nd	nd	PLTP	#	#	PARP-1	#	#	AZU1	0.10	ns
TNF	nd	nd	REG3A	#	#	SLAMF7	#	#	EGFR	−0.02	ns
TSLP	nd	nd	SOD1	#	#	STK4	#	#	EPHB4	#	#
BDNF	Ϯ	Ϯ	UMOD	#	#	TNFRSF10A	#	#	NT-Pro-BNP	#	#

Pearson correlations (r) between NPX values in plasma and serum samples from the total cohort (n = 22) are shown. p values were adjusted by the Holm-Bonferroni (H-B) multiple comparison test; ns, not significant (adj. p > 0.05). nd, not detected. ^#^Excluded due to too many missing values. Ϯ, removed due to technical issue.

**Figure 2:**
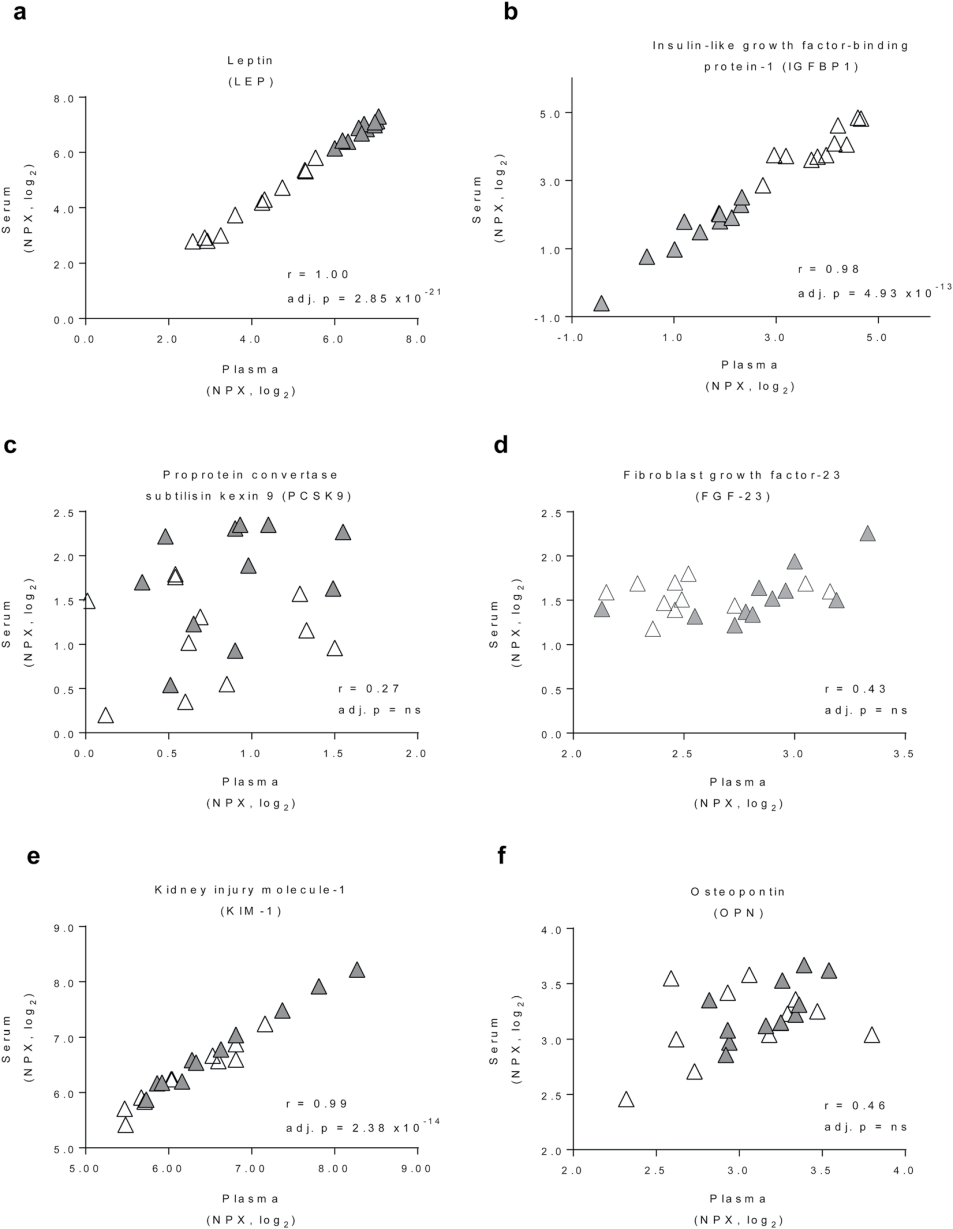
Protein correlations in plasma versus serum. Pearson correlations (r) between normalized protein expression (NPX) values for proteins in plasma and serum samples. Each data point is from one individual (open triangles: obese; closed triangles: lean). p values were adjusted by the Holm-Bonferroni multiple comparison test.

We observed good correlations between plasma and serum samples for leptin (r = 1.00, adj. p < 0.001) and IGFBP-1 (r = 0.98, adj. p < 0.001), which are proteins that exhibited obesity-associated differences in concentration (Fig. 2a,b). Some proteins showed poor correlations, such as PCSK9 (r = 0.27, ns) and FGF-23 (r = 0.43 and 0.64 in the inflammation and cardiovascular II panels, respectively, both ns) (Fig. 2c,d). PCSK9 binds to the receptor for low-density lipoprotein and PCSK9 inhibitors are therefore of intense interest to pharmaceutical companies^39,40^. Studies interchangeably measure PCSK9 in plasma^41,42^ and serum^43,44^, but our result indicates that the choice of biofluid could potentially have a significant impact on the conclusions drawn. Our panels also included the FDA-approved biomarkers KIM-1 and osteopontin, which are used to monitor kidney disease^45,46^. KIM-1 was well correlated between plasma and serum (r = 0.99, adj. p < 0.001) but osteopontin displayed a poor correlation (r = 0.46, ns) (Fig. 2e,f).

### Lipids in plasma versus serum, and in lean versus obese groups

**Figure 3:**
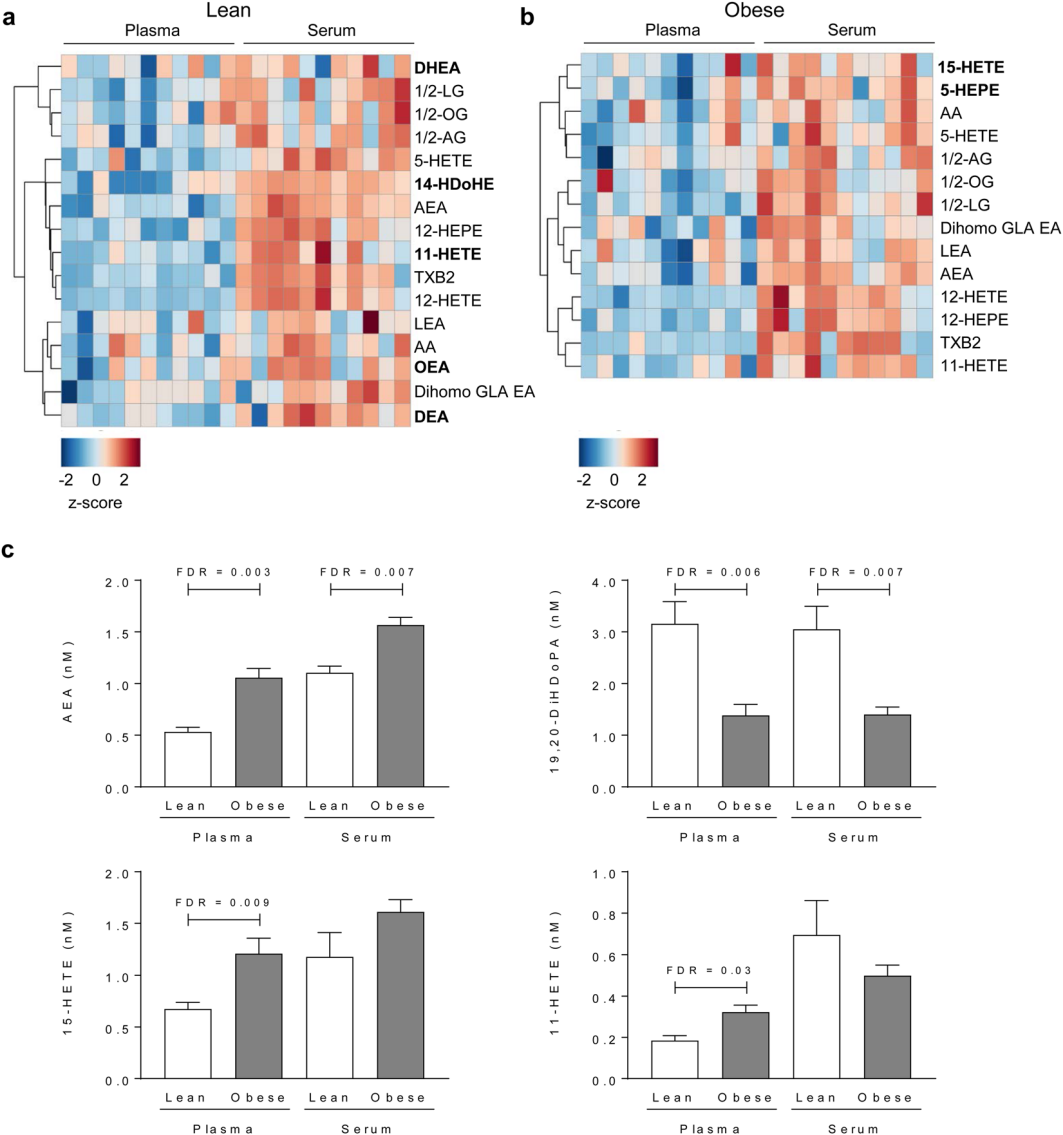
Oxylipins in plasma versus serum, and in lean versus obese groups. Heatmaps showing lipids that exhibited significantly different concentrations in plasma versus serum in (**a**) lean subjects (n = 11) and (**b**) obese subjects (n = 11) after adjustment for multiple comparisons using the false discovery rate (FDR) test at adj. p < 0.05. Lipids that are significantly different in only one of the groups (lean or obese) are marked in bold. Relative lipid concentrations are reported as z-scores. (**c**) Lipids that showed significantly different concentrations between the obese and lean groups in plasma and/or serum after FDR adjustment.

We also performed targeted lipidomics of inflammation-related lipids in plasma and serum from the lean and obese groups. Of the 76 lipids analyzed (see Supplementary Table S2), two were excluded as they did not survive the cut-off criteria for the comparative analysis (Supplementary Fig. S4). For most of the lipids, there were no major differences in concentration between plasma and serum (Supplementary Fig. S5). We observed that concentrations of 21.6% of the lipids in the lean cohort and 18.9% of the lipids in the obese cohort were significantly higher in serum than in plasma (after FDR adjustment, adj. p < 0.05); none of the lipids showed lower concentrations in serum (Fig. 3a,b). In total, 73 lipids survived the cut-off for the correlation analyses; we observed significant correlations between plasma and serum for 64% of the analyzed lipids when analyzed in the lean and obese groups combined (Table 4), and fewer significant correlations were seen when dividing the cohort into obese and lean (Supplementary Table S5**)**.

**Table 4:** Correlations of lipid concentrations in plasma versus serum in all subjects.

Lipid	r	H-B Adj. p value
9,10-DiHOME	0.99	1.78E-18
9,10-DiHODE	0.99	9.18E-18
13-HODE	0.99	2.59E-15
12,13-DiHOME	0.99	3.83E-15
15,16-DiHODE	0.98	4.77E-13
19,20-DiHDoPA	0.97	1.26E-12
9-HOTE	0.97	3.93E-12
9-HODE	0.97	1.18E-11
13-HOTE	0.96	1.48E-10
12(13)-EpOME	0.96	2.04E-10
15(16)-EpODE	0.94	7.71E-09
DHA	0.93	1.60E-08
C16:1n7	0.93	3.84E-08
EPA	0.90	5.81E-07
C18:2n6	0.90	6.28E-07
C14:0	0.89	1.40E-06
AA	0.89	1.83E-06
C18:1n9	0.89	2.17E-06
C12:0	0.88	2.80E-06
DHEA	0.88	2.94E-06
C18:1n7	0.87	5.36E-06
ALA	0.87	7.22E-06
C14 Ceramide	0.87	8.78E-06
C18:3n3	0.85	2.07E-05
C20:5n3	0.84	4.24E-05
PGF2a	0.84	4.75E-05
LEA	0.84	5.45E-05
NA-Gly	0.83	9.19E-05
aLEA	0.83	9.19E-05
LA	0.82	0.0001
C16 Ceramide	0.82	0.0001
C24 dihydroceramide	0.81	0.0002
AEA	0.79	0.0005
1/2-LG	0.78	0.0008
17,18-DiHETE	0.75	0.002
C18:1 Ceramide	0.75	0.002
C24 Ceramide	0.75	0.002
C15:0	0.75	0.002
9(10)-EpOME	0.72	0.005
C18 Ceramide	0.71	0.007
1/2-AG	0.70	0.01
C17:0	0.68	0.01
11,12-DiHETrE	0.67	0.02
C16:0	0.67	0.02
4-HDoHE	0.66	0.02
C20 Ceramide	0.64	0.04
1/2-OG	0.64	0.04
C16:1n7t	0.59	ns
14,15-DiHETrE	0.59	ns
Dihomo GLA EA	0.56	ns
C20:1n9	0.55	ns
C20:2n6	0.54	ns
5-HETE	0.53	ns
12(13)-Ep-9-KODE	0.51	ns
5-HEPE	0.50	ns
DEA	0.49	ns
TXB2	0.45	ns
9c	0.44	ns
15-HETE	0.39	ns
OEA	0.37	ns
18:1 Sphingosine	0.32	ns
12-HEPE	0.31	ns
5,6-DiHETrE	0.27	ns
C20:4n6	0.25	ns
C20:3n6	0.19	ns
9,10-e-DiHO	0.13	ns
C18:0	0.12	ns
11-HETE	0.10	ns
12-HETE	0.08	ns
NO-Gly	0.04	ns
C20:0	0.04	ns
9-KODE	0.01	ns
9,10-EpO	−0.27	ns
C22:4n6	#	#
C22:5n3	#	#
14-HDoHE	#	#

Pearson correlations (r) between lipid concentrations in plasma and serum samples from the total cohort (n = 22) are shown. p values were adjusted by the Holm-Bonferroni (H-B) multiple comparison test; ns, not significant (adj. p > 0.05). ^#^Excluded due to too many missing values.

Four lipids showed significantly different concentrations between the obese and lean groups in plasma and/or serum (Fig. 3c). Concentrations of AEA and 19,20-DiHDoPA were significantly different (higher for AEA and lower for 19,20-DiHDoPA in the obese group) in both plasma and serum, but concentrations of 15-HETE and 11-HETE were significantly different (both higher in the obese group) only in plasma (Fig. 3c).

### Concluding remarks

In this study, we investigated whether the use of plasma or serum would yield different results when screening for obesity-related biomarkers. For most of the proteins and lipids, their concentrations showed good correlations between plasma and serum. However, it is important to note that PCSK9 concentrations did not correlate between plasma and serum, indicating that caution must be taken when comparing studies that use different biofluids. Although most of the protein and lipids had similar concentrations in plasma and serum, those that did differ were generally present at higher concentrations in serum. Importantly, we observed significantly higher concentrations of the key disease-associated biomarker PAI-1 in the obese group only in plasma and not in serum, despite the protein showing higher detectability in serum. This result highlights that sensitivity does not necessarily parallel detectability. Furthermore, some obesity-induced changes, for example of MCP-3 concentrations, were only detected in serum. Collectively, these findings show that care should be taken when choosing biofluids for the study of biomarkers, particularly those for which we report differences in sensitivity/detectability between plasma and serum.

## Methods

### Study participants

We recruited obese subjects [body mass index (BMI) 35–55 kg/m^2^, aged 18–65 years] from a cohort scheduled to undergo gastric bypass surgery, as well as age- and sex-matched lean subjects (BMI 18.5–24.9 kg/m^2^). Subjects were excluded if they were taking anti-inflammatory and/or immunosuppressive drugs, currently smoked, or had been diagnosed with significant gastrointestinal disease or inflammatory bowel disease. Study participants were enrolled in accordance with the Helsinki Declaration and provided written informed consent. The study was approved by the Gothenburg Ethical Review Board #682-14 (ClinicalTrials.gov NCT02322073).

### Blood collection

Venous blood samples obtained from study participants after an overnight fast were collected in eitherplasma tubes spray coated with K_2_EDTA (Greiner Bio One) or serum tubes containing inert separator gel and silica particles as clot activator (Greiner Bio One). Plasma samples were centrifuged immediately whereas serum samples were allowed to clot for 30 min at room temperature before centrifugation (10 min at room temperature, 3,000 rpm Hettich EBA200). Samples were snap-frozen in liquid nitrogen and stored at −80 °C until analysis.

### Multiplex protein assay

Protein biomarkers were analyzed using the proximity extension assay, using four protein panels (inflammation, cardiometabolic, cardiovascular II and cardiovascular III) (Olink Proteomics, Uppsala, Sweden) at the Clinical Biomarkers Facility at Science for Life Laboratory (Uppsala University, Sweden) according to the manufacturer’s instructions. Briefly, 1 µl plasma or serum was incubated with a mixture of 92 proximity antibody pairs tagged with oligonucleotides in a 96-well plate. In this assay, once a pair of antibodies binds to their corresponding antigens in close proximity, linked oligonucleotides hybridize into double stranded DNA, which is further extended and amplified, and ultimately quantified by high-throughput real-time PCR (BioMark™ HD System, Fluidigm Corporation). To avoid intra-assay variability, plasma and serum samples were analyzed on the same plate.

### ELISA

Plasma and serum PAI-1 levels were measured using a commercially available ELISA for Human Total Serpin E1/PAI-1 (#DY9387-05, R&D), according to the manufacturer’s instructions. To ensure that the protein was quantified within the linear range of the standard curve, plasma and serum were diluted 1:100 and 1:500, respectively.

### Measurements of oxylipins, endocannabinoids and ceramides

Oxylipins, endocannabinoids, and ceramides in plasma and serum were isolated and quantified using modifications of published protocols^47–49^. Briefly, plasma or serum aliquots (40 µl) were spiked with deuterated oxylipin, endocannabinoid and ceramide surrogates, mixed with butylated hydroxyl toluene and ethylene diamine tetraacetic acid, and extracted with 200 µl isopropanol containing the internal standards 1-cyclohexyl ureido, 3-dodecanoic acid and 1-phenyl ureido 3-hexanoic acid in isopropanol. The homogenate was then centrifuged (10 min, 4 °C, 15,000 *g*) and the isopropanol supernatant was collected and stored at −20 °C until analysis.

Analytes were separated using a Waters Acquity ultra-performance liquid chromatography (UPLC; Waters, Milford, MA) on a 2.1 mm × 150 mm, 1.7 µm BEH C18 column (Waters) for analysis of oxylipins and endocannabinoids, and 2.1 mm × 150 mm, 1.7 µm BEH C8 column (Waters) for analysis of ceramides. Separated analytes were detected by tandem mass-spectrometry, using electrospray ionization with multi reaction monitoring on an API 6500 QTRAP (Sciex, Redwood City, CA) for oxylipins and endocannabinoids, and an API 4000 QTRAP (Sciex) for ceramides. Analytes were quantified using internal standard methods and 7–9 point calibration curves of authentic standards.

### Measurement of non-esterified fatty acids

Non-esterified fatty acids in plasma and serum were isolated and converted to fatty acid methyl esters (FAMEs) as previously reported^47^. Briefly, plasma or serum aliquots (50 µl) were spiked with lipid class surrogates, mixed with 410 µl isopropanol, followed by 520 µl cyclohexane and 570 µl 0.1 M ammonium acetate. Samples were then centrifuged (5 min, 4 °C, 15,000 *g*), the upper organic phase was collected, and the remainder was re-extracted with a second 520 µl cyclohexane aliquot. The samples were then dried by vacuum centrifugation and reconstituted in 100 µl toluene and 180 µl methanol. To prepare FAMEs, 280 µl of toluene/methanol extracts were enriched with 20 µl methanol containing 60 µM C15:1n5 and incubated with 45 µl 2 M TMS-diazomethane in hexane (Sigma-Aldrich, St. Louis MO) for 30 min at room temperature. Samples were dried under vacuum and the residue was dissolved in 100 µl hexane containing 4 µM C23:0, which acted as an internal standard. Samples were then stored at −20 °C until analysis.

FAMEs were separated on a 30 m × 0.25 mm × 0.25 µm DB-225 ms column in a 6890 gas chromatogram interfaced with a 5973A mass selective detector (Agilent Technologies, Santa Clara, CA). All fatty acids were quantified against a 7-point calibration curves of authentic standards. Peak identifications were based on retention times and m/z ratios, with peak confirmation by inspection of simultaneously acquired full scan spectra collected from 50–400 m/z. Calibrants and internal standards were purchased from NuchekPrep (Elysian, MN), Sigma-Aldrich, or Avanti Polar Lipids. Data were quantified using Chemstation vE.02.14 (Agilent Technologies) against 6–8 point calibration curves.

### Statistical analysis

Data are reported for proteins and lipids that had <30% missing values in: (1) at least one of the four groups (lean plasma, lean serum, obese plasma, obese serum) for the comparative analyses or (2) all of the four groups for the plasma-serum correlations.

Statistical analysis of the protein multiplex data was done in the R environment (version 3.5.1) using packages gplots (3.0.1) and gdata (2.18.0)^50^. For the proteins reported, missing values were replaced with limit of detection (LOD) values. Hierarchical clustering with Pearson correlation distance and complete linkage confirmed that the dataset did not include outliers. Concentrations of proteins are reported as normalized protein expression (NPX) values, an arbitrary unit on a log_2_ scale. Heatmaps were generated using hierarchical clustering based on correlation distance and Ward’s (ward.D2) clustering. Comparisons of protein levels using Student’s t-test were paired when comparing individual donor plasma versus serum values and unpaired when comparing the lean versus obese groups; p values were adjusted for multiple comparisons using either the stringent Holm-Bonferroni test or the commonly used false discovery rate (FDR) test as indicated (adjusted p values < 0.05 were considered significant). Pearson coefficient of correlation (r) values were calculated and p values were adjusted by the Holm-Bonferroni multiple comparison test.

The pathway enrichment analysis for proteins was done using Metascape^51^. Briefly, Gene IDs corresponding to significantly altered proteins were analysed, using the 325 unique proteins that survived the cut-off criteria as the background list. A Gene Ontology category was deemed significantly enriched if the p value was lower than 0.01 and displayed a minimum enrichment of 1.5.

Statistical analysis of the lipidomics data was done in MetaboAnalyst^52^. For the lipids reported, missing values were replaced with half of the lowest reported value. Fatty acid data normalization was optimized in Jmp Pro v 12.0 and confirmed using the Shapiro-Wilk normality test. For statistical analysis, data points underwent log transformation and pareto scaling. Heatmaps were generated using hierarchical clustering based on Euclidean’s method of distance calculation and Ward’s clustering. Unadjusted p values were adjusted using FDR (adjusted p values < 0.05 were considered significant).

## Supplementary information

Supplementary figures and table descriptions

Supplementary Table S1.

Supplementary Table S2.

Supplementary Table S3.

Supplementary Table S4.

Supplementary Table S5.

## Data Availability

Protocols used to generate the findings of this study are available from the corresponding author upon request. In accordance with Swedish ethical regulations and GDPR, primary data from human subjects cannot be made publically available.
