# Supplementary material for: Comparative analysis of obesity-related cardiometabolic and renal biomarkers in human plasma and serum
Source: Sci Rep. 2019 Oct 28;9:15385. doi: 10.1038/s41598-019-51673-0 (PMC6817872; doi:10.1038/s41598-019-51673-0)
Supplement: Supplementary file 1 — Supplementary figures and table descriptions [file 41598_2019_51673_MOESM1_ESM.pdf]

SUPPLEMENTARY INFORMATION

Comparative analysis of obesity-related cardiometabolic and renal biomarkers in human plasma and serum

Meenu Rajan, [1,2,16] Matus Sotak, [1,2,16] Fredrik Barrenäs,[1-3] Tong Shen,[4] Kamil Borkowski,[4] Nicholas J. Ashton,[2,5-7] Christina Biörserud,[9] Tomas L. Lindahl,[10] Sofia Ramström,[10,11] Michael Schöll,[2, 5, 8] Per Lindahl,[1] Oliver Fiehn,[4] John W. Newman,[4,12,13] Rosie Perkins,[1] Ville Wallenius,[9] Stephan Lange,[1,14] Emma Börgeson[1,2,15,17]*

[1] Department of Molecular and Clinical Medicine, Wallenberg Laboratory, Institute of Medicine, University of Gothenburg, Sweden

[2] Wallenberg Centre for Molecular and Translational Medicine, University of Gothenburg, Sweden

[3] Department of Cell & Molecular Biology, Uppsala University, Sweden

[4] NIH West Coast Metabolomics Center, Genome Center, University of California Davis, USA

[5] Department of Psychiatry and Neurochemistry, Institute of Physiology and Neuroscience, University of Gothenburg, Sweden

[6] King's College London, Institute of Psychiatry, Psychology & Neuroscience, Maurice Wohl Clinical Neuroscience Institute, London, UK

[7] NIHR Biomedical Research Centre for Mental Health & Biomedical Research Unit for Dementia at South London & Maudsley NHS Foundation, London, UK

[8] Dementia Research Centre, Institute of Neurology, University College London, UK

[9] Department of Gastrosurgical Research and Education, Institute of Clinical Sciences, Sahlgrenska University Hospital, University of Gothenburg, Sweden

[10] Department of Clinical Chemistry and Department of Clinical and Experimental Medicine, Linköping University, Sweden

[11] Cardiovascular Research Centre, School of Medical Sciences, Örebro University, Sweden

[12] Department of Nutrition, University of California Davis, USA

[13] USDA, ARS, Western Human Nutrition Research Center, Davis, USA

[14] Division of Cardiology, School of Medicine, University of California San Diego, USA

[15] Department of Clinical Physiology, Sahlgrenska University Hospital, Sweden

[16] These authors contributed equally to the work
[17] Lead Contact

***correspondence:** emma.borgeson@wlab.gu.se

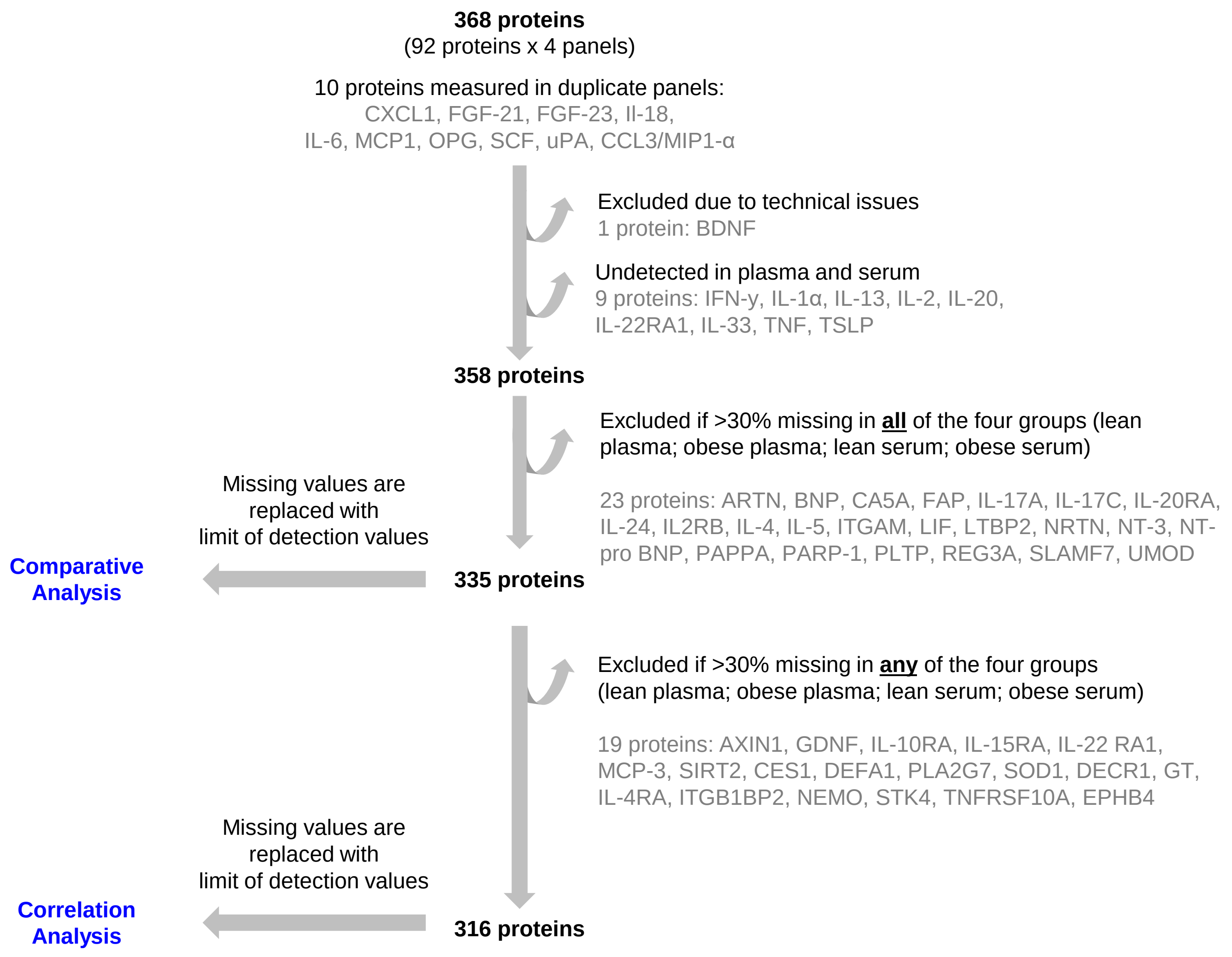

Supplementary Figure S1. Inclusion/exclusion of proteins in the comparative and correlation analyses. Four multiplex panels were selected on the basis of their relevance to obesity-related diseases. Of the 368 proteins analyzed in the four multiplex panels (10 of which were measured in duplicate panels), one protein was excluded due to technical issues, and 9 proteins were excluded because they were undetectable in plasma and serum. For the comparative analysis, 23 additional proteins were excluded because values were missing in >30% of the samples in all of the four groups (lean plasma, lean obese, obese plasma, obese serum). For the correlation analysis, a further 19 proteins were excluded because values were missing in >30% of the samples in any of the four groups. For proteins that passed the cut-off criteria, any missing values were replaced with limit of detection values for that protein.

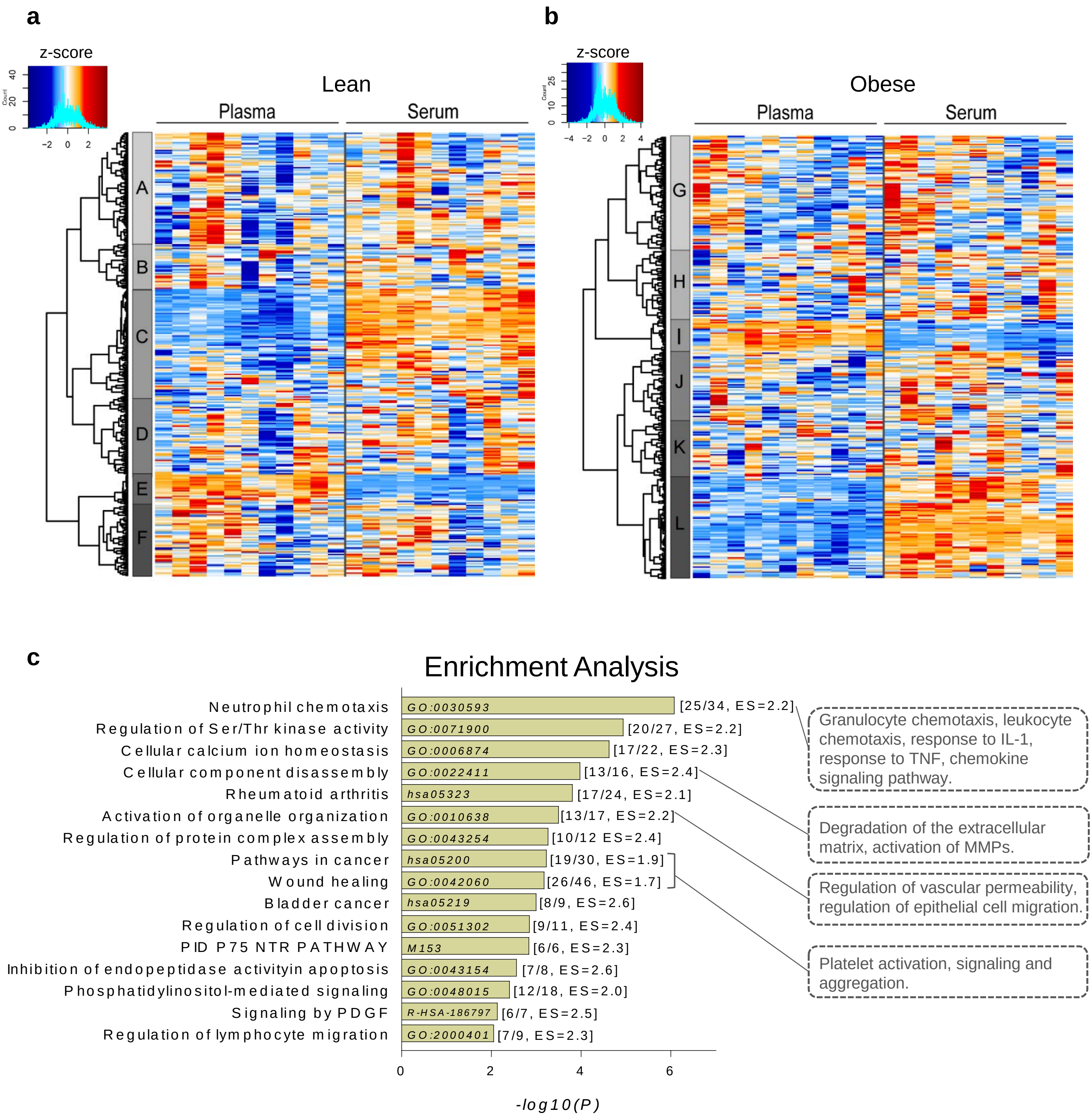

Supplementary Figure S2. Detectability of proteins in plasma versus serum. Heatmaps showing concentrations of protein biomarkers in plasma versus serum in (a) lean subjects (n = 11) and (b) obese subjects (n = 11). Relative protein concentrations are reported as z-scores. (c) Enrichment analysis of the proteins that exhibited significantly different concentrations in plasma versus serum as shown in **Figure 1**.

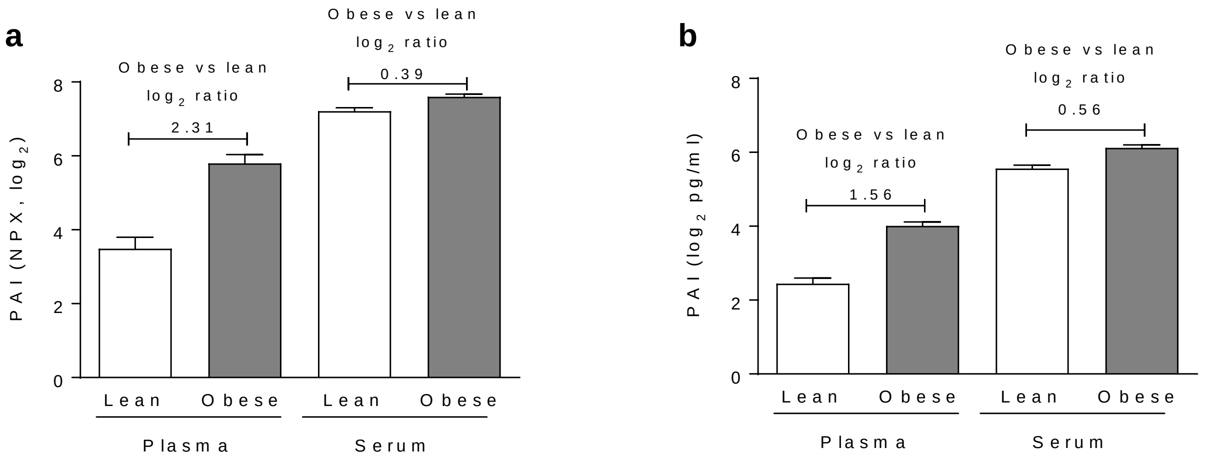

Supplementary Figure S3. Obesity-induced increase in PAI-1 concentrations in plasma and serum. PAI-1 concentrations in plasma and serum from lean and obese subjects (n = 11 per group) as detected by (a) the cardiovascular III panel of the proximity extension assay and (b) ELISA. Data are presented as mean ± SEM. In (a), values are reported as normalized protein expression (NPX) values, an arbitrary unit on a $\log_2$ scale. For comparison, ELISA values in (b) are presented on a $\log_2$ scale.

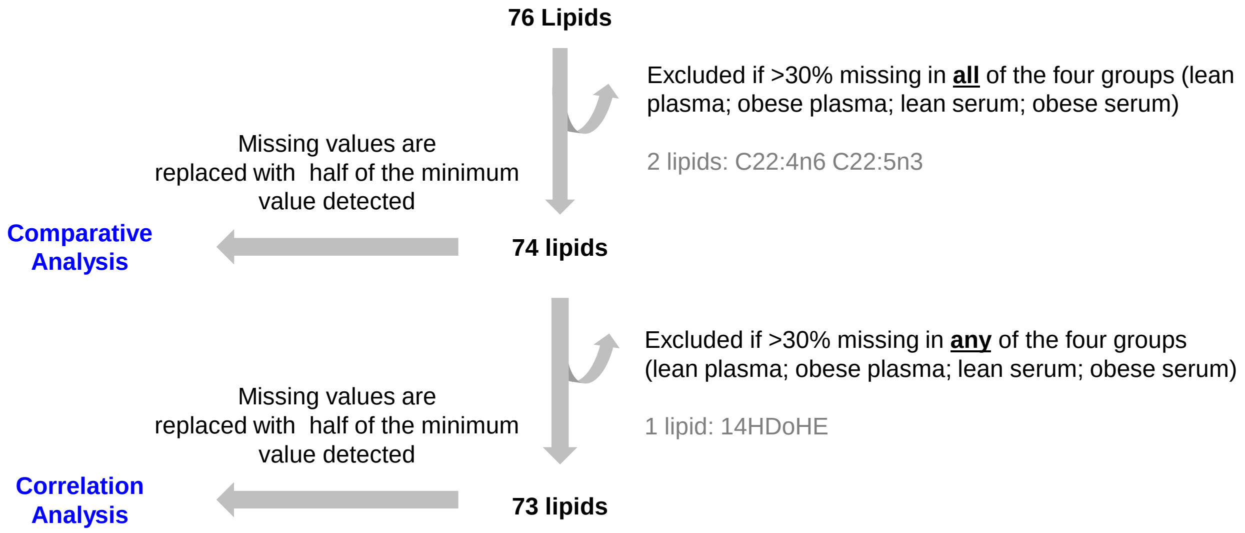

Supplementary Figure S4. Inclusion/exclusion of lipids in the comparative and correlation analyses. Of the 76 lipids analyzed, two were excluded from the comparative analysis because values were missing in >30% of the samples in all of the four groups (lean plasma, lean obese, obese plasma, obese serum) and one further lipid was excluded from the correlation analysis because values were missing in >30% of the samples in any of the four groups. For lipids that passed the cut-off criteria, any missing values were replaced with half of the minimum value detected for that lipid.

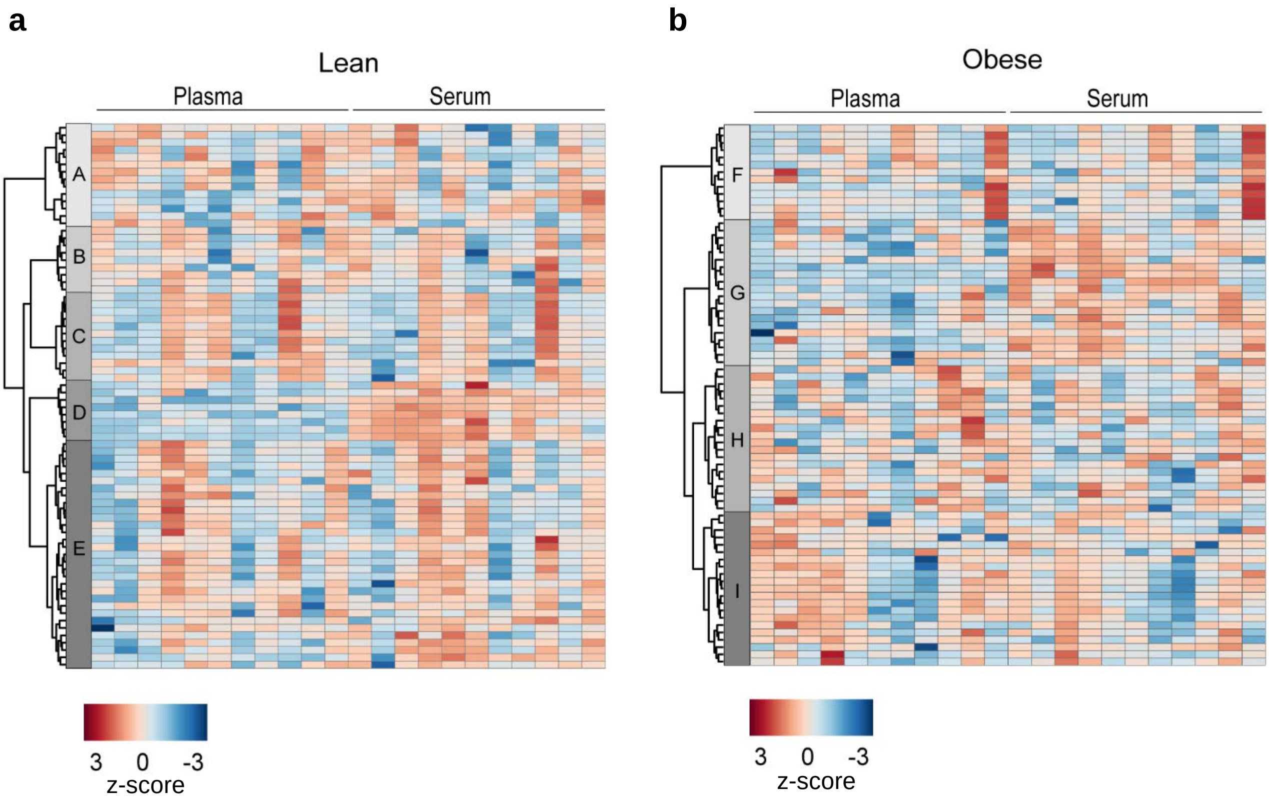

Supplementary Figure S5. Detectability of lipids in plasma versus serum. Heatmaps showing concentrations of lipids in plasma versus serum in (a) lean subjects (n = 11) and (b) obese subjects (n = 11).

Supplementary Table S1. The full list and names of the proteins analyzed in the four multiplex panels: inflammation, cardiometabolic, cardiovascular II and cardiovascular III. The number of missing values for respective protein is noted and highlighted if values were missing in >30% of the samples of the groups.

Supplementary Table S2. The full list and names of the lipids analyzed. The number of missing values for respective lipid is noted and highlighted if values were missing in >30% of the samples of the groups.

Supplementary Table S3. Metascape result from the pathway enrichment analysis of proteins that differed significantly between plasma and serum. The 325 unique proteins that survived the cut-off criteria comprise the background list. A Gene Ontology category was deemed significantly enriched if the p value was lower than 0.01 and displayed a minimum enrichment of 1.5.

Supplementary Table S4. Correlations of protein concentrations in plasma versus serum in lean and obese individuals. Proteins that showed significant correlations in Table 3 are shown and are ordered on the basis of difference in correlation between lean and obese (from highest to lowest). p values were adjusted by the Holm-Bonferroni multiple comparison test.

Supplementary Table S5. Correlations of lipid concentrations in plasma versus serum in lean and obese individuals. Lipids that showed significant correlations in Table 4 are shown and are ordered on the basis of difference in correlation between lean and obese (from greatest to smallest). p values were adjusted by the Holm-Bonferroni multiple comparison test.
